# Effects of Gut Microbiome and Short-Chain Fatty Acids (SCFAs) on Finishing Weight of Meat Rabbits

**DOI:** 10.3389/fmicb.2020.01835

**Published:** 2020-08-11

**Authors:** Shaoming Fang, Xuan Chen, Xiaoxing Ye, Liwen Zhou, Shuaishuai Xue, Qianfu Gan

**Affiliations:** ^1^College of Animal Science, Fujian Agriculture and Forestry University, Fuzhou, China; ^2^College of Life Science, Fujian Agriculture and Forestry University, Fuzhou, China

**Keywords:** gut microbiome, SCFAs, metagenomic, 16S rRNA gene, finishing weight, meat rabbits

## Abstract

Understanding how the gut microbiome and short-chain fatty acids (SCFAs) affect finishing weight is beneficial to improve meat production in the meat rabbit industry. In this study, we identified 15 OTUs and 23 microbial species associated with finishing weight using 16S rRNA gene and metagenomic sequencing analysis, respectively. Among these, butyrate-producing bacteria of the family Ruminococcaceae were positively associated with finishing weight, whereas the microbial taxa related to intestinal damage and inflammation showed opposite effects. Furthermore, interactions of these microbial taxa were firstly found to be associated with finishing weight. Gut microbial functional capacity analysis revealed that CAZymes, such as galactosidase, xylanase, and glucosidase, could significantly affect finishing weight, given their roles in regulating nutrient digestibility. GOs related to the metabolism of several carbohydrates and amino acids also showed important effects on finishing weight. Additionally, both KOs and KEGG pathways related to the membrane transportation system and involved in aminoacyl-tRNA biosynthesis and butanoate metabolism could act as key factors in modulating finishing weight. Importantly, gut microbiome explained nearly 11% of the variation in finishing weight, and our findings revealed that a subset of metagenomic species could act as predictors of finishing weight. SCFAs levels, especially butyrate level, had critical impacts on finishing weight, and several finishing weight-associated species were potentially contributed to the shift in butyrate level. Thus, our results should give deep insights into how gut microbiome and SCFAs influence finishing weight of meat rabbits and provide essential knowledge for improving finishing weight by manipulating gut microbiome.

## Introduction

The gut microbiome comprises the collective genome of the trillions of microorganisms colonizing the intestinal tract, which is responsible for vital metabolic, immunological, and nutritional functions (Sidhu and van der Poorten, 2017). Short-chain fatty acids (SCFAs), mainly acetate, propionate, and butyrate, are recognized as important metabolites derived from the fermentation of indigestible dietary fibers by gut microbial species, which play fundamental roles in maintaining intestinal homeostasis and energy metabolism regulation (Priyadarshini et al., 2018).

Recently, the roles of gut microbiome and SCFAs in modulating growth performances of farm animals have been extensively studied. For instance, Wang J. et al. (2019) indicated that certain bacteria of the family Ruminococcaceae, the genus *Faecalibacterium*, and the genus *Prevotella* are involved in metabolizing dietary fibers which subsequently affect SCFA production, which exerted important roles in feed intake and body weight modulation in pigs. Du et al. (2018) demonstrated that both butyrate-producing bacteria *Butyricimonas* and the digestive enzyme family metabolic pathway could potentially affect the body weight of beef calves. Additionally, research on the gut microbiome of broiler chickens suggested that increased abundance of *Lactobacillus* and *Bifidobacterium*, and facilitated generations of lactate and SCFAs, had a positive influence on feed efficiency and body weight gain (Yu et al., 2019).

Finishing weight is regarded as a crucial growth trait of meat rabbits, which directly affects broiler rabbits’ meat production. However, only few studies have unraveled the relationship between the gut microbiome and finishing weight of rabbits. Zeng *et al.* reported that changes in the abundance of YS2, *Bacteroides*, *Lactococcus*, *Lactobacillus*, and *Prevotella* in the gut microbial communities of rabbits were associated with finishing weight (Zeng et al., 2015). Wang Q. et al. (2019) found that *Coprococcus* and *Ruminococcaceae_UCG-004* may hinder the production of pro-inflammatory factors that exert beneficial effects on the finishing weight of rabbits. Moreover, North et al. (2019) indicated that rabbits with greater abundances of Eubacteriaceae, Natranaerobiaceae, Peptococcaceae, and Syntrophomonadaceae in the gut microbial communities tended to gain more weight at finishing. Nevertheless, these investigations are based on 16S rRNA gene sequencing analysis, which cannot determine species and functional capacities that affect finishing weight. Additionally, the potential roles of SCFAs in modulating the finishing weight of meat rabbits remain unknown. Hence, the present study aimed to assess the effects of gut microbiome and SCFAs on finishing weight using 16S rRNA gene sequencing, metagenomic sequencing, and fecal SCFA level data. We not only identified a number of microbial taxa and metabolic functions associated with finishing weight but also unraveled the correlations between SCFA levels and finishing weight. In addition, we evaluated the role of the gut microbiome in the phenotypic variation of finishing weight. Such findings could improve our understanding of how the gut microbiome and SCFAs modulate finishing weight and thus provide important information for the meat rabbit industry.

## Materials and Methods

### Experimental Rabbits and Sample Collection

One hundred and five Ira rabbits (28 ± 2 days, 53 males and 52 females) with similar weaning weight were randomly distributed to separated cages (one rabbit per cage) and fed with the fatten diet (details are shown in Supplementary Table S1) until finishing (72 ± 2 days). Finishing body weight was measured, and hard fecal samples were collected. Finishing weights were sorted, and the top 5 rabbits with the highest body weight (high group) and the bottom 5 with the lowest body weight (low group) were selected for metagenomic sequencing. All rabbits were healthy and had not received antibiotics, anti-coccidial drugs, probiotics, or prebiotics during the experimental period. All fecal samples were snap frozen in liquid nitrogen for transportation and stored at −80°C until further processing.

### 16S rRNA Gene Sequencing

Microbial genomic DNA of fecal samples was extracted by the QIAamp Fast DNA Stool Mini Kit (QIAGEN, Germany) according to the manufacturer’s instructions. The purity and integrity of total DNA was detected by using the NanoDrop ND-2000 spectrophotometer (Thermo Fisher Scientific, United States) and 1.5% agarose gel electrophoresis, respectively. Hypervariable regions V3–V4 of the 16S ribosomal RNA gene were then amplified by using the primer 341F:CCTACGGGNGGCWGCAG and 806R:GGACTACHVGGGTATCTAAT and sequenced by the HiSeq 2500 platform (Illumina, United States) according to the manufacturer’s manuals. QIIME (v.1.9.1) was used for the quality control process of sequencing data including filtering out of the primers, barcodes, and low-quality sequences (quality score < 20) (Caporaso et al., 2010). High-quality paired-end reads were merged into tags by using FLASH (v.1.2.11) (Magoc and Salzberg, 2011). We rarefied the library size of microbial sequences to 40,000 tags per sample to normalize the sequencing depth (Fu et al., 2015). USEARCH (v.10.0) was used to cluster tags into operational taxonomic units (OTUs) at 97% sequence similarity (Edgar, 2010). OTUs which had relative abundance < 0.1% and were presented in less than 1% of the experimental rabbits were filtered out before further analysis. OTU taxonomic category assignments were performed by using the SILVA database (v.132) (Quast et al., 2013).

### Two-Part Model Analysis

After sex and cage rearing effect corrections, residuals of finishing weight phenotypic values were used for association analysis between finishing weight and relative abundances of OTUs by using a two-part model method as described before (Fu et al., 2015). In brief, the two-part model analysis was consisted of binary, quantitative, and meta models. The binary model describes a binomial analysis that assesses for the effect of the presence/absence of the gut microbe on finishing weight. The quantitative model tests for the association between finishing weight and the abundance of each OTU, but only the samples where that microbe is present were included in the analysis. The meta model was used to integrate the effect of both binary and quantitative analysis. The final association *p*-value was assigned from the minimum of *p*-values from the binary analysis, quantitative analysis, and meta-analysis. Skewness correction was performed by 1000 × permutation tests. False discovery rate (FDR)-adjusted *p* < 0.05 was set as the significance threshold.

### Metagenomic Sequencing

A paired-end (PE) DNA library was constructed for each sample according to the manufacturer’s instruction (Illumina, United States), and sequencing was performed on an Illumina HiSeq 4000 platform. Quality control, adapter trimming, and low-quality read filtering of raw data were performed by using fastp (v.0.19.4) (Chen et al., 2018) and obtained an average of 64,042,801 high-quality reads for each sample. We assembled the high-quality reads into contigs using the MEGAHIT (v.1.1.3) (Li et al., 2015). The contigs with more than 200 bp in length were used for open reading frame (ORF) prediction with MetaGeneMark (v.2.10) (Zhu et al., 2010). A non-redundant gene catalog was established by removing the redundant genes from the predicted ORFs using Cd-hit (v.4.6.1) (Fu et al., 2012). The gene abundance profile was generated by mapping the high-quality reads against the non-redundant gene catalog using MOCAT (v2.0) (Kultima et al., 2016). DIAMOND (v.0.9.24) (Buchfink et al., 2015) was used for taxonomic category and GO term assignments of the predicted genes by aligning against the non-redundant (NR) database and Gene Ontology (GO) database, respectively. Carbohydrate-Active enZYmes (CAZymes) were annotated by hmmscan program in HMMER (v.3.1) (Eddy, 2011). GhostKOALA was used to extract KEGG Orthology (KO) and KEGG pathway annotation information from the Kyoto Encyclopedia of Genes and Genomes (KEGG) database (Kanehisa et al., 2016).

### Co-abundance Group (CAG) Analysis

In order to construct CAG, we first calculated the correlation coefficients among the finishing weight-associated microbial taxa using the Sparse Correlations for Compositional data (SparCC) algorithm (Friedman and Alm, 2012). Then, CAGs were defined by Ward’s linkage hierarchical clustering method and PERMANOVA (999 permutations, *p* < 0.05) based on the SparCC correlation coefficient matrix through made4 and vegan R packages, respectively (Zhang et al., 2016). The CAG interaction networks were established based on FDR-adjusted *p* < 0.05 and | r| > 0.35 by CYTOSCAPE (v.3.7.0) (Shannon et al., 2003). Spearman’s correlation analysis with FDR correction was performed to compute the association between CAGs and finishing weight.

### Estimating Phenotypic Variance Explained by the Gut Microbiome

To evaluate the contribution of the gut microbiome to the variation of finishing weight, we performed a 100× cross-validation (Fu et al., 2015). The data set was randomly divided into an 80% discovery data set and a 20% validation data set. In the discovery data set, we performed two-part model analysis to identify a number of (n) OTUs that were significantly associated with finishing weight at a certain *p*-value and assessed the effect sizes of binary and quantitative features (β_1_ and β_2_) of each OTUs. In the validation data set, the effect of the gut microbiome on finishing weight (r_m_) was estimated by an additive model: r_m_ = $\sum_{j=1}^{\text{n}}\left(\beta_{1}+b_{\text{j}}+\beta_{2}\,q_{\text{j}}\right)$, b_j_ and q_j_ corresponding to the binary and quantitative feature of j OTU, respectively. We calculated the squared correlation coefficient (R^2^) between r_m_ and the phenotypic value, which represents d or the phenotypic variance explained by the gut microbiome. To ensure validity and stability of the estimation, we repeated the cross-validation for 100 times and calculated the average value of the explained variations.

### Determinations of SCFA in Fecal Samples

The concentrations of SCFAs were measured in the fecal samples using gas chromatography on an Agilent 7890A GC system with a flame ionization detector (Agilent Technologies, United States) according to the previously described method with some modifications (Reddivari et al., 2017). Hundred and fifty milligram fecal samples were homogenized in 1 ml of water –0.5% phosphoric acid by Vortex-Genie 2 (Scientific Industries, United States). The mixture was centrifuged at 15,000 rpm for 10 min at 4°C. The supernatant (600 μl) which was mixed with an equal volume of ethyl acetate was used for filtering by a 0.35 mm filter membrane and then transferred to a glass gas chromatography vial prior to injection into a GC instrument. The internal standard of 2-methyl butyrate at a final concentration of 10 mM was added to the organic phase to correct injection variability among the samples. 10–100 mM acetate, 1–10 mM propionate, and 1–10 mM butyrate were set as external standards, and five sample intervals were prepared to calculate retention times and create standard curves. The concentrations of SCFAs in fecal samples were determined by comparing to standard curves. The correlations between finishing weight-associated species and SCFA levels were analyzed by using the Spearman method.

### Basic Statistical Analysis

The Wilcoxon rank-sum test with FDR correction was used to identify the metagenomic species and function capacities of the gut microbiome having significantly different enrichments between high and low finishing rabbits. To identify important species that could predict finishing weight variation, random forest analysis was performed by using species relative abundance data, and a predictor species was determined based on the mean decrease in accuracy of discrimination > 2 (Breiman, 2001). Two-sided unpaired Student’s *t*-test with FDR correction was performed to detect the differences in levels of SCFAs in fecal samples between high and low finishing weight rabbits. Except the differential metagenomic species which were visualized online by Interactive Tree Of Life (iTOL) (Letunic and Bork, 2019), the other plots were accomplished in R software.

## Results

### Gut Microbiota Associated With Finishing Weight

The phylogenetic analysis of gut microbiota in experimental rabbits has been previously described (Fang et al., 2019, 2020). Here, we focused on identifying the gut microbiota associated with finishing weight. The phenotypic distribution of finishing weight in experimental rabbits is shown in Supplementary Figure S1A. The phenotypic value of rabbits in the high finishing weight group was significantly different than that in the low finishing weight group (FDR adjusted *P* < 0.05, Supplementary Figure S1B).

**FIGURE 1:**
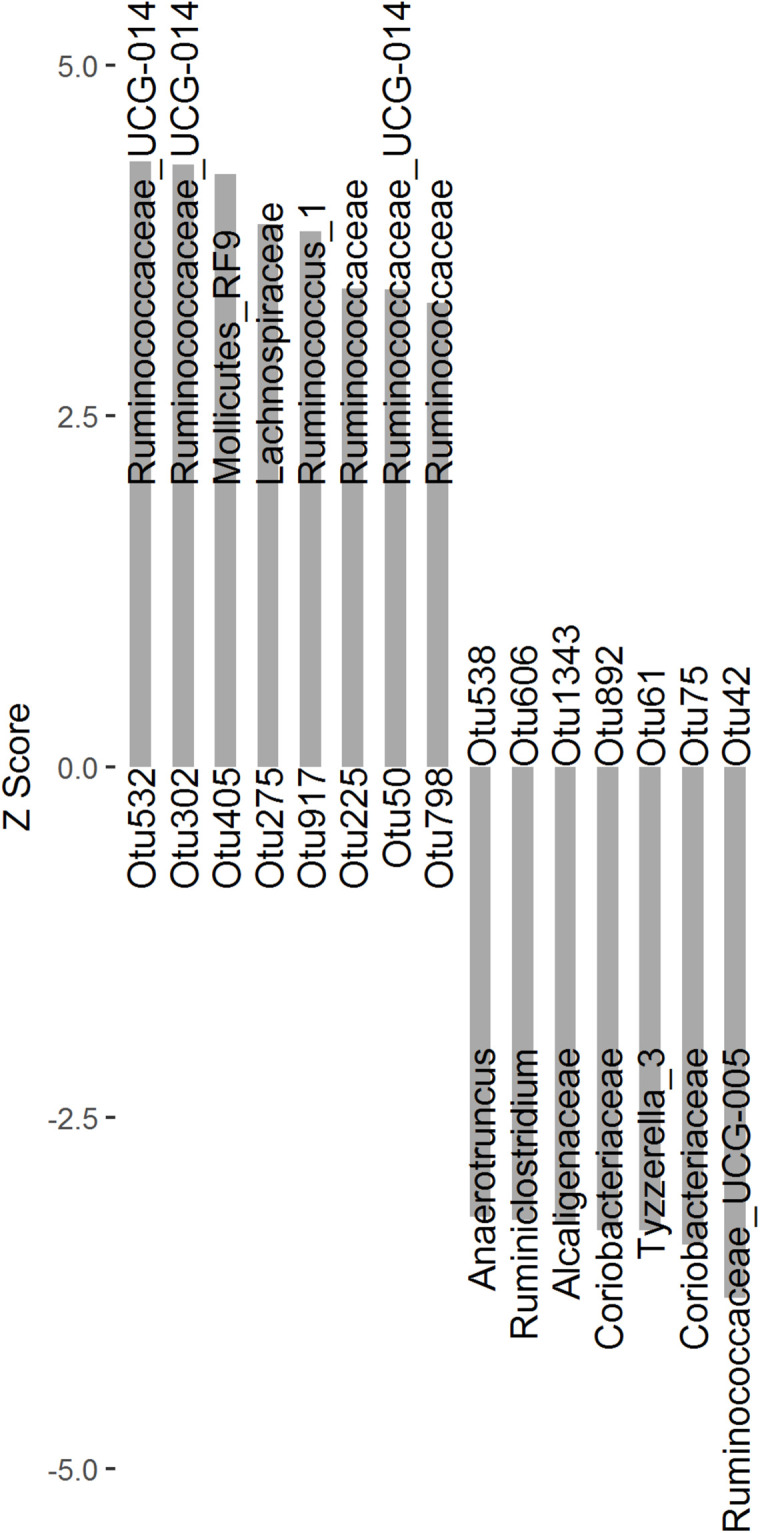
The OTUs significantly associated with finishing weight (FDR adjusted *P* < 0.05) are shown as Z scores.

To identify microbial taxa associated with finishing weight, we performed a two-part model analysis using the relative abundances of OTUs and phenotypic values in the experimental population. We identified 15 OTUs associated with finishing weight (Figure 1 and Supplementary Table S2, FDR adjusted *P* < 0.05), of which 8 OTUs exhibited positive associations and the remaining showed negative associations. Among the OTUs positively associated with finishing weight, one was annotated to the order Mollicutes_RF9. Two were annotated to the family Ruminococcaceae, and one to the family Lachnospiraceae. Three OTUs were annotated to the genus *Ruminococcaceae_UCG-014*, and one to the genus *Ruminococcus_1*. As for the OTUs negatively associated with finishing weight, three OTUs were annotated to the family level (two belonging to the family Coriobacteriaceae and one to the family Alcaligenaceae) and four to the genus level (*Ruminococcaceae_UCG-005*, *Tyzzerella_3*, *Ruminiclostridium*, and *Anaerotruncus* one OTU each).

**FIGURE 2:**
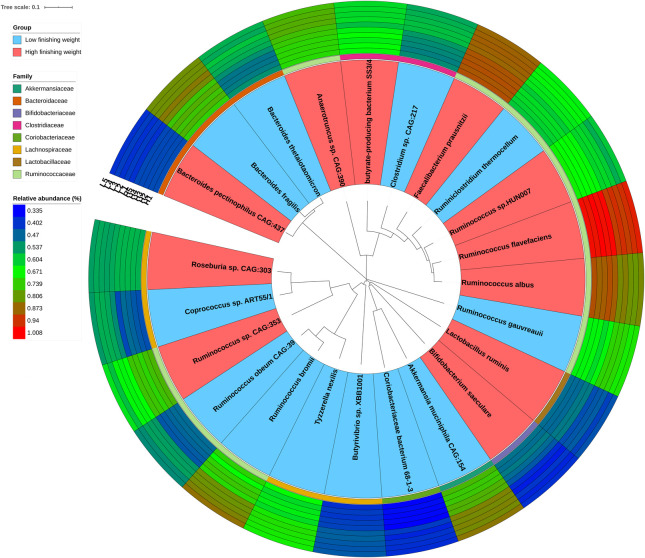
The phylogenetic relationships and abundances of metagenomic species enriched in high and low finishing weight rabbits. The phylogenetic tree of species is shown as the innermost layers. Labels with red and blue color represent high and low finishing weight groups, respectively. The different color strips in the third layer correspond to different families as indicated by the color code on the left. The outermost layers depict the relative abundances of differential species between high and low finishing weight individuals.

Due to the great limitations of 16S rRNA gene sequencing in both resolution and accuracy at the species level, we performed metagenomic sequencing of five individuals with the highest and lowest phenotypic values, respectively. We used the Wilcoxon rank-sum test with metagenomic sequencing data to determine microbial species showing different enrichments between high and low finishing weight rabbits. A total of 23 species were identified (Figure 2 and Supplementary Table S3, FDR adjusted *P* < 0.05), including four species from the family Ruminococcaceae *Ruminococcus* sp. *CAG:353*, *Ruminococcus albus*, *Ruminococcus* sp. *HUN007*, and *Ruminococcus flavefaciens*, which were abundant in rabbits with high finishing weight. In addition, *Faecalibacterium prausnitzii*, *Bifidobacterium saeculare*, *Roseburia* sp. *CAG:303*, *Lactobacillus ruminis*, *butyrate-producing bacterium SS3/4*, and *Bacteroides pectinophilus CAG:437* were also enriched in rabbits with high finishing weight. Conversely, we found that *Ruminococcus gauvreauii*, *Ruminococcus bromii*, and *Ruminococcus obeum CAG:39* (members of family Ruminococcaceae) were augmented in rabbits with low finishing weight. Additionally, *Akkermansia muciniphila CAG:154*, *Butyrivibrio* sp. *XBB1001*, *Bacteroides fragilis*, *Clostridium* sp. *CAG:217*, *Coprococcus* sp. *ART55/1*, *Bacteroides thetaiotaomicron*, *Ruminiclostridium thermocellum*, *Tyzzerella nexilis*, *Coriobacteriaceae bacterium 68-1-3*, and *Anaerotruncus* sp. *CAG:390* were also abundant in rabbits with low finishing weight.

### Interactions of Microbial Taxa Correlated With Finishing Weight

**FIGURE 3:**
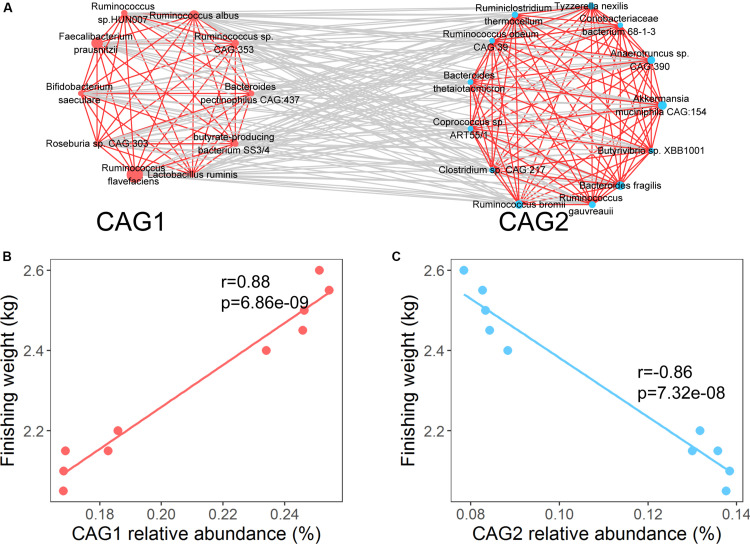
Interactions among the species responding for finishing weight. **(A)** The interaction network of finishing weight-associated species. The red nodes represent positively associated species and the blue nodes represent negatively associated species. The node size indicates the average relative abundance of each species. Lines linked to nodes indicate significant correlations among the species (FDR adjusted *P* < 0.05, |r| > 0.45), with red and gray colors showing positive and negative correlations, respectively. The species are clustered into two co-abundance groups (CAGs) by PERMANOVA at P < 0.05. **(B)** The association of the abundance of CAG 1 with finishing weight. **(C)** The association of the abundance of CAG 2 with finishing weight.

Previous studies have demonstrated that the interactions of gut microbes play vital roles in host physiology, development, and growth (Ramayo-Caldas et al., 2016; Ke et al., 2019). To investigate the potential effect of interactions among the identified species on finishing weight, we constructed a co-abundance network based on SparCC correlation coefficients. The co-abundance network was consisted of two co-abundance groups (CAGs; Figure 3A and Supplementary Figure S2). The species that were positively associated with finishing weight were clustered into CAG 1 through intra-positive interactions, whereas CAG 2 was formed by intra-positive interactions among the species that were negatively associated with finishing weight. In addition, CAG 1 had positive associations with finishing weight, but CAG 2 was negatively correlated with finishing weight (Figures 3B,C). Notably, when we analyzed the entire experimental population, the co-abundance network could also be established using finishing weight associated OTUs, and similar correlations were observed between the CAGs and finishing weight (Supplementary Figure S3).

### Functionalities of the Gut Microbiome Related to Finishing Weight

Functionalities of the gut microbiome related to finishing weight were detected by comparing the abundances of CAZymes, GOs, and KEGG items between rabbits with high and low finishing weights.

**FIGURE 4:**
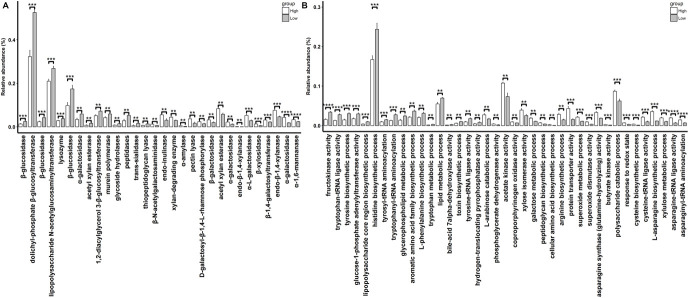
The differential CAzymes **(A)** and GOs **(B)** identified to associate with finishing weight. “**”, “***”, and “****” represents for FDR adjusted *P* < 0.01, *P* < 0.005, and *P* < 0.001, respectively.

A total of 30 CAZymes with significantly different abundances in rabbits with high and low finishing weights were identified (Figure 4A and Supplementary Table S4, FDR adjusted *P* < 0.05). Among these, 16 CAZymes especially galactosidase and xylanase (e.g., GH27, GH95, GH4, GH51, GH120, and GH11) were significantly enriched in the gut microbiome of rabbits with high finishing weight. 14 CAZymes, mainly glucosidase (e.g., GH30, GH1, and GH3) had significantly higher abundances in the gut microbiome of low finishing weight rabbits.

We also identified 39 GOs that showed different abundances between high and low finishing weight rabbits (Figure 4B and Supplementary Table S4, FDR-adjusted *P* < 0.05). Twenty-two out of 39 GOs were more abundant in rabbits with high finishing weight, most of which were related to the metabolism of xylan, galactose, and arabinose (e.g., GO:0005997, GO:0006012, GO:0009045, and GO:0019572) and to the biosynthetic processes of asparagine, cysteine, and arginine (e.g., GO:0006421, GO:0004816, GO:0070981, GO:0004066, GO:0004817, GO:0019344, and GO:0006526). The remaining 17 GOs, predominant in rabbits with low finishing weight, were correlated with the metabolism of glucose (e.g., GO:0008865 and GO:0008878), tryptophan, tyrosine, and phenylalanine (e.g., GO:0004830, GO:0006571, GO:0006436, GO:0006437, GO:0009094, and GO:0006568).

**FIGURE 5:**
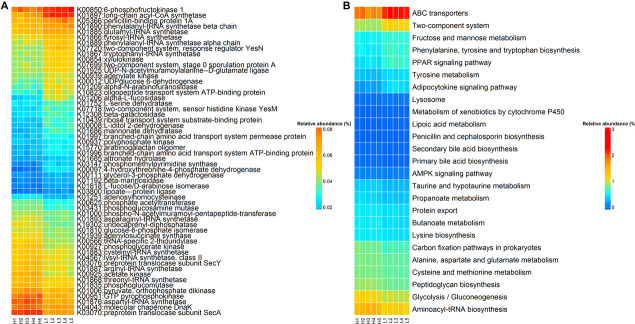
The KEGG function terms showing different enrichments between high and low finishing weight rabbits. **(A)** Heat map of KOs showing different enrichments between high and low finishing weight rabbits. The *X*-axis shows the sample IDs, e.g., H1 representing the individual 1 with high finishing weight. **(B)** Heat map of KEGG pathways showing different enrichments between high and low finishing weight rabbits.

Sixty KOs exhibited distinct abundances in rabbits with high and low finishing weight (Figure 5A and Supplementary Table S4, FDR-adjusted *P* < 0.05). Twenty-six KOs were more abundant in high finishing weight rabbits, most of which were correlated with butanoate metabolism (e.g., K00171, K00169, and K03737), aminoacyl-tRNA biosynthesis (e.g., K01883, K01893, and K01876), and the cysteine and methionine metabolism (e.g., K00016, K00812, and K11358). The other 34 KOs related to tyrosine metabolism (e.g., K04072, K13954, K00274, and K18933), fructose and mannose metabolism (e.g., K16370, K01818, K02771, and K00879), ABC transporters (e.g., K10038, K05668, K19309, and K10554), and two-component system (e.g., K06596, K11614, K03412, and K01034) were plentiful in low finishing weight rabbits.

On the other hand, 25 differential enriched KEGG pathways were identified in the gut microbiome of rabbits with distinct finishing weights (Figure 5B and Supplementary Table S4, FDR adjusted *P* < 0.05). Similarly, we found that aminoacyl-tRNA biosynthesis, glycolysis/gluconeogenesis, butanoate metabolism, and cysteine and methionine metabolism were more active in the gut microbial communities of high finishing weight rabbits. Meanwhile, in rabbits with low finishing weight, gut microbiota were more capable of operating ABC transporters and two-component system; additionally, the metabolism of tyrosine, fructose, and mannose was more active in these animals.

### Gut Microbiome as a Predictor of Finishing Weight Variation

**FIGURE 6:**
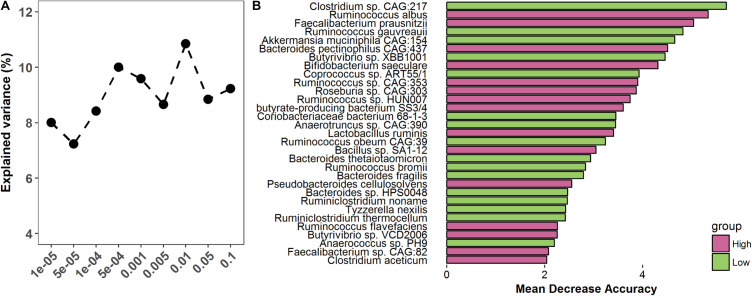
The predictive role of the gut microbiome in finishing weight. **(A)** The variation of finishing weight explained by the gut microbiome at different *P*-values. **(B)** Metagenomic species were identified as being able to predict finishing weight.

To assess whether the gut microbiome was able to predict finishing weight, we investigated how much degree of phenotypic variance of finishing weight was explained by the gut microbiome by performing 100 times cross-validation analyses at different *p*-value thresholds (ranging from 10^–5^ to 0.1) using the OTU data. The OTUs identified at *p* = 1 × 10^–5^ could explain 8.01% of the variations in finishing weight (Figure 6A); at *p* = 0.1, the explained variation increased to 10.85%, given that more OTUs were included in the analysis as the threshold increased.

Then, to evaluate whether a subset of metagenomic species within the gut microbial community could predict the finishing weights of rabbits, we performed random forest analysis in high and low finishing weight rabbits. Thirty-one species were identified as being able to predict finishing weight and showed a substantial overlap with the species above identified as being associated with finishing weight (Figure 6B).

### Changes in Fecal SCFAs Linked to Finishing Weight

**FIGURE 7:**
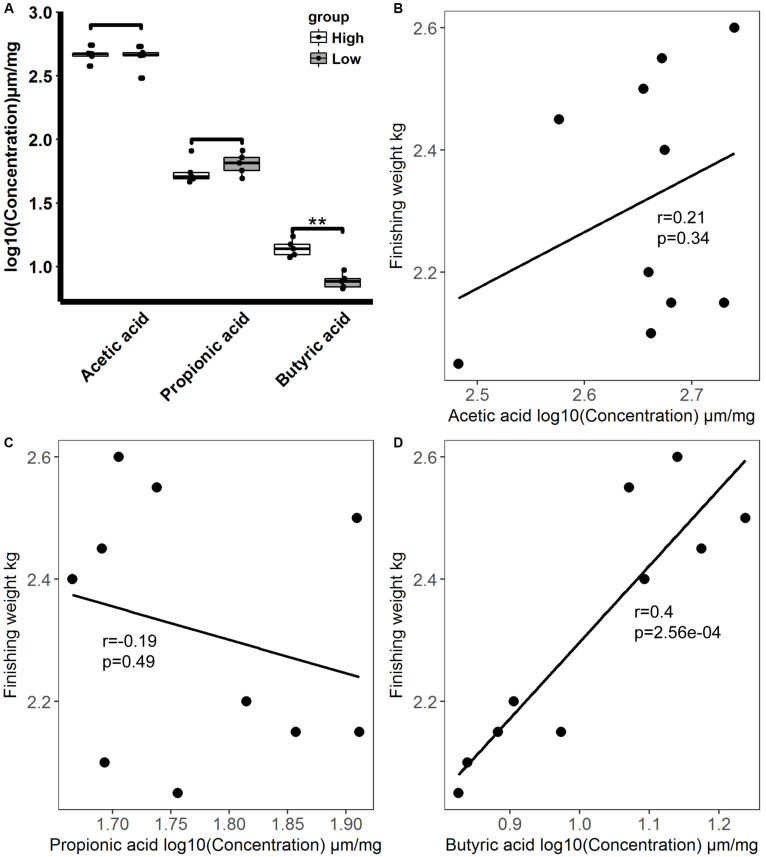
Fecal short-chain fatty acid levels in high and low finishing weight rabbits and their correlations with finishing weight. **(A)** Comparisons of fecal short-chain fatty acid levels between high and low finishing weight rabbits. **(B–D)** Correlations between fecal short-chain fatty acid levels and finishing weight.

As SCFAs produced by gut microbiota exert important effects on host energy metabolism and intestinal health status regulation in animals (den Besten et al., 2013), we analyzed the levels of acetic acid, propionic acid, and butyric acid in the feces of rabbits with high and low finishing weights (Figure 7A). Our results showed that the level of butyric acid was significantly greater in the feces of rabbits with high finishing weight in comparison to that in rabbits with low finishing weight (FDR adjusted *P* < 0.05), but the levels of propionic acid tended to decrease. No significant change in the levels of acetic acid was observed between high and low finishing weight rabbits. In addition, correlation analysis indicated that the level of butyric acid had a significantly positive association with finishing weight, while the levels of acetic acid and propionic acid showed the tendency of positive and negative association with finishing weight, respectively (Figures 7B–D).

**FIGURE 8:**
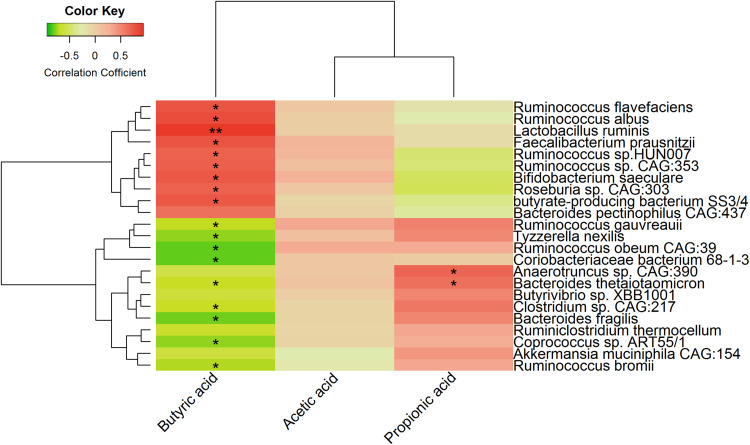
Heat map showing correlations between finishing weight-associated species and SCFAs levels.

We also analyzed the correlations between finishing weight-associated species and SCFAs levels. As shown in Figure 8, nine out of ten species enriched in the high finishing weight individuals were positively associated with the level of butyric acid, and nine out of thirteen species augmented in the low finishing weight individuals were negatively correlated with the level of butyric acid. Both *Anaerotruncus* sp. *CAG:390* and *Bacteroides thetaiotaomicron* were more abundant in low finishing weight rabbits and had positive associations with the level of propionic acid. However, no significant correlations were observed between the level of acetic acid and finishing weight-associated species.

## Discussion

Accumulating evidence indicates that the gut microbiome plays a pivotal role in nutrient metabolism, energy utilization, and health maintenance in domestic animals (O’Callaghan et al., 2016). Accordingly, the gut microbiome is considered to be an essential factor that affects growth of livestock animals. Thus, identifying gut microbiota as key for production performances could be a game changer in the livestock production industry (Maltecca et al., 2020). SCFAs are important metabolites generated by gut microbial fermentation of complex carbohydrates, which have emerged as key regulators in intestinal health and energy homeostasis regulation. However, the effects of gut microbiome and SCFAs on finishing weight of meat rabbits have received far less attention. Here, we systematically and comprehensively evaluated how the gut microbiome and SCFAs affect the finishing weight of meat rabbits.

We identified 15 OTUs that were significantly associated with finishing weight, of which eight OTUs were annotated to members of the family Ruminococcaceae (Figure 1). Among these, *Ruminococcaceae_UCG-014* and *Ruminococcus_1* were positively associated with finishing weight, while *Ruminococcaceae_UCG-005* and *Ruminiclostridium* showed the opposite correlations. *Ruminococcaceae_UCG-014* belongs to butyrate-producing bacteria capable of degrading cellulose and hemicellulose in feeds and has a potential role in maintenance of intestinal health (Dai et al., 2018). *Ruminococcus_1* is another important player in butyrate production by fermenting complex non-digestible polysaccharides, which is considered to be related to intestinal anti-inflammatory responses (Xie et al., 2019). In contrast, *Ruminococcaceae_UCG-005* and *Ruminiclostridium* were positively correlated with diarrhea incidence and intestinal villi damage, respectively (Hung et al., 2019; Xing et al., 2019).

Family Ruminococcaceae encompasses many species. It is important to know which are associated with a good intestinal health status. Metagenomic sequencing analysis sheds light on this. Our results showed that different species from family Ruminococcaceae exert contrasting effects on finishing weight (Figure 2). For example, *Ruminococcus albus* and *Ruminococcus flavefaciens* were enriched in rabbits with high finishing weight, but *Ruminococcus gauvreauii* and *Ruminococcus bromii* were more abundant in low finishing weight individuals. Both *R. albus* and *R. flavefaciens* are well-known cellulolytic bacteria involved in the butyrate metabolic pathway and abundant in ruminants with high body weight gain (Carberry et al., 2012; Mao et al., 2017; Izuddin et al., 2019). However, both *R. gauvreauii* and *R. bromii* are mucolytic bacteria which can induce chronic intestinal inflammation (Lyra et al., 2009; Crost et al., 2018; Fernandez et al., 2018). Moreover, *R. bromii* is negatively associated with body weight in pigs (Tran et al., 2018).

We identified several other species that could significantly affect finishing weight. For instance, *Faecalibacterium prausnitzii* is a major butyrate producer in the intestine, which has an important role in providing energy sources for enterocytes and fulfilling anti-inflammatory actions (Foditsch et al., 2014). *Lactobacillus ruminis* is a member of lactic acid bacteria that has multiple probiotic properties, including inhibition of intestinal pathogens, fortification of epithelial barrier functions, and modulation of immune responses (Yu et al., 2017). In agreement with previous studies (Oikonomou et al., 2013; Gao et al., 2017), we found that these two species were more abundant in the gut microbiome of individuals with a higher body weight. In contrast, although both *Akkermansia muciniphila* and *Bacteroides fragilis* were considered to be promising candidates as probiotics (Gilbert et al., 2013; Zhang T. et al., 2019), the overgrowth of these two species in the gut of rabbits could result in the decreased butyrate yield and lead to the incidence of epizootic rabbit enteropathy (ERE, a severe gastrointestinal syndrome disease) (Jin et al., 2018). Hence, these two species augmenting in the gut microbiome of meat rabbits could be a potential trigger for reduction of finishing weight.

In order to make a microbial community-based inference, it is important not only to identify microbial taxa associated with finishing weight but also to understand the effects of the interactions among such taxa on finishing weight. Thus, co-abundance networks were established at both metagenomic species and OTU level. We found that co-occurrence and co-exclusion relationships exist among finishing weight-associated microbial taxa (Figure 3 and Supplementary Figure S3). Importantly, their interactions which could affect the finishing weight have intimate relations with their distinct roles in host metabolic and intestinal health regulation. Similarly, earlier studies have revealed that interactions of gut microbes affect the body weight in farm animals. Yang et al. demonstrated that the strong co-occurrence relationship between butyrate-producing bacteria (e.g., Ruminococcaceae) and lactic acid bacteria (e.g., *Lactobacillus*) may affect porcine body weight gain by regulating host appetite and feeding behavior (Yang et al., 2018). Zhang et al. (2018) suggested that the significant co-exclusion relationship between Enterobacteriaceae and Prevotellaceae was associated with the metabolism of SCFAs and energy, which provides an important direction for manipulating gut microbiota to improve the body weight in pigs. Additionally, Gao et al. (2017) and Ma et al. (2018) highlighted the important role of gut microbial interactions in improving growth performances of broiler chickens.

Distinct metagenomic functional capacities were also found to be associated with finishing weight. The CAZyme comparison analysis showed that galactosidase and xylanase were more abundant in rabbits with high finishing weight, while glucosidase was more abundant in low finishing weight rabbits (Figure 4A). Adding extra galactosidase and xylanase into feeds has been reported to enhance nutrient (e.g., fibers and amino acids) digestibility and improve the body weight of farm animals (Zhang et al., 2017; Jasek et al., 2018). This could be used to explain why rabbits with a greater abundance of these two enzymes showed greater finishing weights. However, the effects of gut microbial glucosidase on body weight are conflicting. De Cesare et al. (2017) demonstrated that a higher abundance of glucosidase in the gut microbiome contributed to increased body weight gain of broiler chickens, whereas Shokryazdan et al. (2017) suggested that the reduced glucosidase enzyme activity in the intestine led to an increase in body weight in broiler chickens.

Our results also showed that GOs related to xylan, galactose, and arabinose metabolism were more abundant in rabbits with high finishing weight, while those associated with glucose metabolism showed higher abundances in low finishing weight rabbits (Figure 4B). Additionally, we found that the gut microbiota of rabbits with high finishing weight preferred to be involved in the biosynthesis of asparagine, cysteine, and arginine, but gut microbial communities in low finishing weight rabbits were more active in metabolizing tryptophan, tyrosine, and phenylalanine. Although asparagine is a non-essential amino acid, emerging evidence has demonstrated that it plays a key role in attenuating intestinal injury and improving the energy status of enterocytes, all of which are good for the health and growth of animals (Wang et al., 2015; Patra et al., 2019). Cysteine is able to regulate the antioxidant status and expression of anti-inflammatory genes in intestinal cells, which exert beneficial effects on body weight gain in pigs and chickens (Dilger and Baker, 2007; Yang and Liao, 2019). Arginine is a hub precursor for the synthesis of various important metabolic molecules, including NO and polyamines, exerting regulatory roles in the host’s metabolic processes (Flynn et al., 2002). Thus, it has been used to improve meat production in meat-producing animals (Hu et al., 2017; Liu et al., 2019). Conversely, gut microbiota is involved in aromatic amino acids (tryptophan, tyrosine, and phenylalanine) metabolism has been linked to chronic low-grade inflammation and metabolic disorders, which may hinder development and growth of animals (Wang et al., 2011; Agus et al., 2018).

On the other hand, the aminoacyl-tRNA biosynthesis pathway and the metabolic pathways of butanoate, cysteine, and methionine were more active in high finishing weight rabbits, whereas ABC transporters, and fructose, mannose, and tyrosine metabolic pathways were overrepresented in rabbits with low finishing weight (Figure 5). The aminoacyl-tRNA biosynthesis pathway is an essential metabolic function of microorganisms and is intimately correlated with maturation of gastrointestinal microbiota (Zhang K. et al., 2019). More importantly, microbial mature status is fundamental for host development and growth (Wang X. et al., 2019). Several studies have demonstrated that the gut microbial butanoate metabolic pathway exerts beneficial effects on body weight of livestock due to its potential impact on increasing energy intake, improving intestinal histomorphometric characteristics (e.g., villi height and crypt depth), and modulating the immune system (Ndou et al., 2018; Che et al., 2019; Jacquier et al., 2019). Similar to the abovementioned cysteine, methionine is crucial in maintaining the functions of intestinal cells by regulating the redox status (Azad et al., 2019). Thus, enhanced methionine metabolism in the gut microbial communities contributed to bovine body weight gain (Jiao et al., 2017). On the other hand, ABC transporters involved in transporting a large variety of sterols, lipids, drugs, and primary and secondary metabolites (Hou et al., 2017) have recently been related to fat deposition, drug resistance, and colonic inflammation (Andersen et al., 2015; Fang et al., 2017), which may do harm for growth performances in animals. Furthermore, enhanced fructose, mannose, and tyrosine metabolic pathways in the gut microbiome have been associated with host intestinal permeability and metabolic endotoxemia that implies their negative effects on growth of animals (Wang et al., 2011; Do et al., 2018).

To further emphasize the predictive role of the gut microbiome in finishing weight variation, we estimated that the gut microbiome could explain 7.23–10.85% of the variation in finishing weight (Figure 6A), which is similar to host genetics on finishing weight (6.3–12.1%) (Piles et al., 2007). Moreover, we identified 31 species that could act as predictors of finishing weight, most of which are strongly associated with finishing weight (Figure 6B). Consistent with prior studies, our findings suggested that the gut microbiome should be regarded as a key variable of body weight prediction model of farm animals (Wang X. et al., 2019; Wen et al., 2019).

Our results also revealed that the level of fecal butyric acid was significantly higher in rabbits with high finishing weight in comparison to rabbits with low finishing weight (Figure 7A). Importantly, the level of butyric acid showed positive associations with finishing weight (Figure 7B). Butyrate is not only recognized as an essential energy source but also acts as a signal transduction molecule of G-protein-coupled receptors (FFAR3, GPR109A) and as epigenetic regulators of gene expression by inhibiting histone deacetylase (HDAC) (Kasubuchi et al., 2015). Due to these important properties, butyrate exhibits wide energy and metabolic regulation abilities and strong anti-inflammatory effects that positively affect the body weight gain of animals (Bedford and Gong, 2018). Consistently, we found that several microbial species positively associated with finishing weight (such as, *Faecalibacterium prausnitzii*, *Roseburia* sp. *CAG:303*, and *butyrate-producing bacterium SS3/4*) were well-known butyrate producers (Qin et al., 2012) that had positive associations with butyric acid level (Figure 8). Interestingly, we also observed that both *Lactobacillus ruminis* and *Bifidobacterium saeculare* were positively associated with butyric acid level. This is in accordance with previous findings which revealed that high levels of intestinal *Lactobacillus* sp. and *Bifidobacterium* sp. contributed to increased butyrate levels (Le Roy et al., 2015; Kim et al., 2020). In addition, our results showed the changes in the levels of acetic acid and propionic acid in the feces of rabbits with high and low finishing weight and their potential correlations with finishing weight (Figures 7A,C,D). These findings were in agreement with previous studies suggested that both acetate and propionate were important players in modulating growth performances in animals (Liu et al., 2018; Min et al., 2019).

Our study, although limited by a small rabbit population, provides important information that supports the potential of gut microbiota manipulation in improving finishing weight of meat rabbits. However, from potential to action, the next steps are to validate the causative roles of some gut bacterial species in modulating finishing weight through specific pathogen-free (SPF) rabbits’ intervention experiment and to gain more mechanistic insights into the cross talk between gut microbiome and host and its effects on growth performances of meat rabbits by multi-omics studies.

## Conclusion

In conclusion, the current study identified key microbial taxa associated with finishing weight. We also determined the gut microbial CAZymes, GOs, KOs, and KEGG pathways associated with finishing weight. We found that the gut microbiome could act as a key predictor of finishing weight variation. In addition, we emphasized the important effects of SCFAs on finishing weight, especially butyrate. Hence, our findings provide essential insights into how the gut microbiome and SCFAS affect finishing weight and imply that manipulating the gut microbial community could be an efficient strategy to improve finishing weight in the meat rabbit industry.

## Data Availability Statement

The datasets presented in this study can be found in online repositories. The names of the repository/repositories and accession number(s) can be found in the article/ Supplementary Material.

## Ethics Statement

The animal study was reviewed and approved by the Animal Care and Use Committee (ACUC) in Fujian Agriculture and Forestry University.

## Author Contributions

QG designed the experiments, analyzed the data, and wrote and revised the manuscript. SF and XC performed the experiments, analyzed the data, and wrote the manuscript. LZ, XY, and SX performed the experiments. All authors read and approved the final manuscript.

## Conflict of Interest

The authors declare that the research was conducted in the absence of any commercial or financial relationships that could be construed as a potential conflict of interest.

## Supplementary Material

FIGURE S1 Finishing weight phenotypic values of all rabbits **(A)** and high and low individuals **(B)**.

FIGURE S2 The finishing weight associated species formed two clusters using Ward clustering algorithm.

FIGURE S3 **(A,B)** Constructions of CAGs and interactions network by using the finishing weight associated OTUs. **(C,D)** Associations between CAGs and finishing weight.

TABLE S1 Composition of pellet diet for fattening rabbits.

TABLE S2 The OTUs showing significant associations with finishing weight.

TABLE S3 Differential microbial species between high and low finishing weight rabbits.

TABLE S4 Functionalities showing different enrichments between high and low finishing weight rabbits.

## References

[B1] Agus A. Planchais J. Sokol H. (2018). Gut microbiota regulation of tryptophan metabolism in health and disease. *Cell Host Microbe* 23 716–724. 10.1016/j.chom.2018.05.003 29902437

[B2] Andersen V. Svenningsen K. Knudsen L. A. Hansen A. K. Holmskov U. Stensballe A. (2015). Novel understanding of ABC transporters ABCB1/MDR/P-glycoprotein, ABCC2/MRP2, and ABCG2/BCRP in colorectal pathophysiology. *World J. Gastroenterol.* 21 11862–11876. 10.3748/wjg.v21.i41.11862 26557010 PMC4631984

[B3] Azad M. A. K. Liu G. Bin P. Ding S. Kong X. Guan G. (2019). Sulfur-containing amino acid supplementation to gilts from late pregnancy to lactation altered offspring’s intestinal microbiota and plasma metabolites. *Appl. Microbiol. Biotechnol.* 104 1227–1242. 10.1007/s00253-019-10302-6 31853564

[B4] Bedford A. Gong J. (2018). Implications of butyrate and its derivatives for gut health and animal production. *Anim. Nutr.* 4 151–159. 10.1016/j.aninu.2017.08.010 30140754 PMC6104520

[B5] Breiman L. (2001). Random forests. *Mach. Learn.* 45 5–32. 10.1023/A:1010933404324

[B6] Buchfink B. Xie C. Huson D. H. (2015). Fast and sensitive protein alignment using DIAMOND. *Nat. Methods* 12 59–60. 10.1038/nmeth.3176 25402007

[B7] Caporaso J. G. Kuczynski J. Stombaugh J. Bittinger K. Bushman F. D. Costello E. K. (2010). QIIME allows analysis of high-throughput community sequencing data. *Nat. Methods* 7 335–336. 10.1038/nmeth.f.303 20383131 PMC3156573

[B8] Carberry C. A. Kenny D. A. Han S. McCabe M. S. Waters S. M. (2012). Effect of phenotypic residual feed intake and dietary forage content on the rumen microbial community of beef cattle. *Appl. Environ. Microbiol.* 78 4949–4958. 10.1128/AEM.07759-11 22562991 PMC3416373

[B9] Che L. Hu Q. Wang R. Zhang D. Liu C. Zhang Y. (2019). Inter-correlated gut microbiota and SCFAs changes upon antibiotics exposure links with rapid body-mass gain in weaned piglet model. *J. Nutr. Biochem.* 74:108246. 10.1016/j.jnutbio.2019.108246 31671360

[B10] Chen S. Zhou Y. Chen Y. Gu J. (2018). fastp: an ultra-fast all-in-one FASTQ preprocessor. *Bioinformatics* 34 i884–i890. 10.1093/bioinformatics/bty560 30423086 PMC6129281

[B11] Crost E. H. Le Gall G. Laverde-Gomez J. A. Mukhopadhya I. Flint H. J. Juge N. (2018). Mechanistic insights into the cross-feeding of *Ruminococcus gnavus* and *Ruminococcus bromii* on host and dietary carbohydrates. *Front. Microbiol.* 9:2558. 10.3389/fmicb.2018.02558 30455672 PMC6231298

[B12] Dai S. J. Zhang K. Y. Ding X. M. Bai S. P. Luo Y. H. Wang J. P. (2018). Effect of dietary non-phytate phosphorus levels on the diversity and structure of cecal microbiota in meat duck from 1 to 21 d of age. *Poult. Sci.* 97 2441–2450. 10.3382/ps/pey090 29617914

[B13] De Cesare A. Sirri F. Manfreda G. Moniaci P. Giardini A. Zampiga M. (2017). Effect of dietary supplementation with *Lactobacillus acidophilus* D2/CSL (CECT 4529) on caecum microbioma and productive performance in broiler chickens. *PLoS One* 12:e0176309. 10.1371/journal.pone.0176309 28472118 PMC5417446

[B14] den Besten G. van Eunen K. Groen A. K. Venema K. Reijngoud D. J. Bakker B. M. (2013). The role of short-chain fatty acids in the interplay between diet, gut microbiota, and host energy metabolism. *J. Lipid Res.* 54 2325–2340. 10.1194/jlr.R036012 23821742 PMC3735932

[B15] Dilger R. N. Baker D. H. (2007). Oral N-acetyl-L-cysteine is a safe and effective precursor of cysteine. *J. Anim Sci.* 85 1712–1718. 10.2527/jas.2006-835 17371789

[B16] Do M. H. Lee E. Oh M. J. Kim Y. Park H. Y. (2018). High-glucose or -fructose diet cause changes of the gut microbiota and metabolic disorders in mice without body weight change. *Nutrients* 10:639. 10.3390/nu10060761 29899272 PMC6024874

[B17] Du R. Jiao S. Dai Y. An J. Lv J. Yan X. (2018). Probiotic *Bacillus amyloliquefaciens* C-1 improves growth performance, stimulates GH/IGF-1, and regulates the gut microbiota of growth-retarded beef calves. *Front. Microbiol.* 9:2006. 10.3389/fmicb.2018.02006 30210477 PMC6120984

[B18] Eddy S. R. (2011). Accelerated profile HMM searches. *PLoS Comput. Biol.* 7:e1002195. 10.1371/journal.pcbi.1002195 22039361 PMC3197634

[B19] Edgar R. C. (2010). Search and clustering orders of magnitude faster than BLAST. *Bioinformatics* 26 2460–2461. 10.1093/bioinformatics/btq461 20709691

[B20] Fang S. Chen X. Pan J. Chen Q. Zhou L. Wang C. (2020). Dynamic distribution of gut microbiota in meat rabbits at different growth stages and relationship with average daily gain (ADG). *BMC Microbiol.* 20:116. 10.1186/s12866-020-01797-5 32410629 PMC7227296

[B21] Fang S. Chen X. Zhou L. Wang C. Chen Q. Lin R. (2019). Faecal microbiota and functional capacity associated with weaning weight in meat rabbits. *Microb. Biotechnol.* 12 1441–1452. 10.1111/1751-7915.13485 31571427 PMC6801154

[B22] Fang S. Xiong X. Su Y. Huang L. Chen C. (2017). 16S rRNA gene-based association study identified microbial taxa associated with pork intramuscular fat content in feces and cecum lumen. *BMC Microbiol.* 17:162. 10.1186/s12866-017-1055-x 28724349 PMC5518119

[B23] Fernandez J. Moreno F. J. Olano A. Clemente A. Villar C. J. Lombo F. (2018). A galacto-oligosaccharides preparation derived from lactulose protects against colorectal cancer development in an animal model. *Front. Microbiol.* 9:2004. 10.3389/fmicb.2018.02004 30233512 PMC6127505

[B24] Flynn N. E. Meininger C. J. Haynes T. E. Wu G. (2002). The metabolic basis of arginine nutrition and pharmacotherapy. *Biomed. Pharmacother.* 56 427–438. 10.1016/s0753-3322(02)00273-1 12481979

[B25] Foditsch C. Santos T. M. Teixeira A. G. Pereira R. V. Dias J. M. Gaeta N. (2014). Isolation and characterization of *Faecalibacterium prausnitzii* from calves and piglets. *PLoS One* 9:e116465. 10.1371/journal.pone.0116465 25551453 PMC4281123

[B26] Friedman J. Alm E. J. (2012). Inferring correlation networks from genomic survey data. *PLoS Comput. Biol.* 8:e1002687. 10.1371/journal.pcbi.1002687 23028285 PMC3447976

[B27] Fu J. Bonder M. J. Cenit M. C. Tigchelaar E. F. Maatman A. Dekens J. A. (2015). The gut microbiome contributes to a substantial proportion of the variation in blood lipids. *Circ. Res.* 117 817–824. 10.1161/CIRCRESAHA.115.306807 26358192 PMC4596485

[B28] Fu L. Niu B. Zhu Z. Wu S. Li W. (2012). CD-HIT: accelerated for clustering the next-generation sequencing data. *Bioinformatics* 28 3150–3152. 10.1093/bioinformatics/bts565 23060610 PMC3516142

[B29] Gao P. Ma C. Sun Z. Wang L. Huang S. Su X. (2017). Feed-additive probiotics accelerate yet antibiotics delay intestinal microbiota maturation in broiler chicken. *Microbiome* 5:91. 10.1186/s40168-017-0315-1 28768551 PMC5541433

[B30] Gilbert J. A. Krajmalnik-Brown R. Porazinska D. L. Weiss S. J. Knight R. (2013). Toward effective probiotics for autism and other neurodevelopmental disorders. *Cell* 155 1446–1448. 10.1016/j.cell.2013.11.035 24360269 PMC4166551

[B31] Hou Y. P. He Q. Q. Ouyang H. M. Peng H. S. Wang Q. Li J. (2017). Human gut microbiota associated with obesity in chinese children and adolescents. *Biomed. Res. Int.* 2017:7585989. 10.1155/2017/7585989 29214176 PMC5682041

[B32] Hu C. J. Jiang Q. Y. Zhang T. Yin Y. L. Li F. N. Deng J. P. (2017). Dietary supplementation with arginine and glutamic acid modifies growth performance, carcass traits, and meat quality in growing-finishing pigs. *J. Anim. Sci.* 95 2680–2689. 10.2527/jas.2017.1388 28727042

[B33] Hung D. Y. Cheng Y. H. Chen W. J. Hua K. F. Pietruszka A. Dybus A. (2019). *Bacillus licheniformis*-fermented products reduce diarrhea incidence and alter the fecal microbiota community in weaning piglets. *Animals* 9:1145. 10.3390/ani9121145 31847281 PMC6940967

[B34] Izuddin W. I. Loh T. C. Samsudin A. A. Foo H. L. Humam A. M. Shazali N. (2019). Effects of postbiotic supplementation on growth performance, ruminal fermentation and microbial profile, blood metabolite and GHR, IGF-1 and MCT-1 gene expression in post-weaning lambs. *BMC Vet. Res.* 15:315. 10.1186/s12917-019-2064-9 31477098 PMC6719353

[B35] Jacquier V. Nelson A. Jlali M. Rhayat L. Brinch K. S. Devillard E. (2019). Bacillus subtilis 29784 induces a shift in broiler gut microbiome toward butyrate-producing bacteria and improves intestinal histomorphology and animal performance. *Poult. Sci.* 98 2548–2554. 10.3382/ps/pey602 30668816

[B36] Jasek A. Latham R. E. Manon A. Llamas-Moya S. Adhikari R. Poureslami R. (2018). Impact of a multicarbohydrase containing alpha-galactosidase and xylanase on ileal digestible energy, crude protein digestibility, and ileal amino acid digestibility in broiler chickens. *Poult. Sci.* 97 3149–3155. 10.3382/ps/pey193 29897592

[B37] Jiao S. Cao H. Dai Y. Wu J. Lv J. Du R. (2017). Effect of high-fat diet and growth stage on the diversity and composition of intestinal microbiota in healthy bovine livestock. *J. Sci. Food Agric.* 97 5004–5013. 10.1002/jsfa.8380 28417460

[B38] Jin D. X. Zou H. W. Liu S. Q. Wang L. Z. Xue B. Wu (2018). The underlying microbial mechanism of epizootic rabbit enteropathy triggered by a low fiber diet. *Sci. Rep.* 8 12489. 10.1038/s41598-018-30178-2 30131509 PMC6104036

[B39] Kanehisa M. Sato Y. Kawashima M. Furumichi M. Tanabe M. (2016). KEGG as a reference resource for gene and protein annotation. *Nucleic Acids Res.* 44 D457–D462. 10.1093/nar/gkv1070 26476454 PMC4702792

[B40] Kasubuchi M. Hasegawa S. Hiramatsu T. Ichimura A. Kimura I. (2015). Dietary gut microbial metabolites, short-chain fatty acids, and host metabolic regulation. *Nutrients* 7 2839–2849. 10.3390/nu7042839 25875123 PMC4425176

[B41] Ke S. Fang S. He M. Huang X. Yang H. Yang B. (2019). Age-based dynamic changes of phylogenetic composition and interaction networks of health pig gut microbiome feeding in a uniformed condition. *BMC Vet. Res.* 15:172. 10.1186/s12917-019-1918-5 31126262 PMC6534858

[B42] Kim H. Jeong Y. Kang S. You H. J. Ji G. E. (2020). Co-culture with *Bifidobacterium catenulatum* improves the growth, gut colonization, and butyrate production of *Faecalibacterium prausnitzii*: in vitro and in vivo studies. *Microorganisms* 8:788. 10.3390/microorganisms8050788 32466189 PMC7285360

[B43] Kultima J. R. Coelho L. P. Forslund K. Huerta-Cepas J. Li S. S. Driessen M. (2016). MOCAT2: a metagenomic assembly, annotation and profiling framework. *Bioinformatics* 32 2520–2523. 10.1093/bioinformatics/btw183 27153620 PMC4978931

[B44] Le Roy C. I. Stsepetova J. Sepp E. Songisepp E. Claus S. P. Mikelsaar M. (2015). New insights into the impact of Lactobacillus population on host-bacteria metabolic interplay. *Oncotarget* 6 30545–30556. 10.18632/oncotarget.5906 26437083 PMC4741550

[B45] Letunic I. Bork P. (2019). Interactive Tree Of Life (iTOL) v4: recent updates and new developments. *Nucleic Acids Res.* 47 W256–W259. 10.1093/nar/gkz239 30931475 PMC6602468

[B46] Li D. Liu C. M. Luo R. Sadakane K. Lam T. W. (2015). MEGAHIT: an ultra-fast single-node solution for large and complex metagenomics assembly via succinct de Bruijn graph. *Bioinformatics* 31 1674–1676. 10.1093/bioinformatics/btv033 25609793

[B47] Liu B. Wang W. Zhu X. Sun X. Xiao J. Li D. (2018). Response of gut microbiota to dietary fiber and metabolic interaction with SCFAs in piglets. *Front. Microbiol.* 9:2344. 10.3389/fmicb.2018.02344 30323803 PMC6172335

[B48] Liu S. Tan J. Hu Y. Jia X. Kogut M. H. Yuan J. (2019). Dietary l-arginine supplementation influences growth performance and B-cell secretion of immunoglobulin in broiler chickens. *J. Anim. Physiol. Anim. Nutr.* 103 1125–1134. 10.1111/jpn.13110 31155767

[B49] Lyra A. Rinttila T. Nikkila J. Krogius-Kurikka L. Kajander K. Malinen E. (2009). Diarrhoea-predominant irritable bowel syndrome distinguishable by 16S rRNA gene phylotype quantification. *World J. Gastroenterol.* 15 5936–5945. 10.3748/wjg.15.5936 20014457 PMC2795180

[B50] Ma Y. Wang W. Zhang H. Wang J. Zhang W. Gao J. (2018). Supplemental *Bacillus subtilis* DSM 32315 manipulates intestinal structure and microbial composition in broiler chickens. *Sci. Rep.* 8:15358. 10.1038/s41598-018-33762-8 30337568 PMC6194052

[B51] Magoc T. Salzberg S. L. (2011). FLASH: fast length adjustment of short reads to improve genome assemblies. *Bioinformatics* 27 2957–2963. 10.1093/bioinformatics/btr507 21903629 PMC3198573

[B52] Maltecca C. Bergamaschi M. Tiezzi F. (2020). The interaction between microbiome and pig efficiency: a review. *J. Anim. Breed Genet.* 137 4–13. 10.1111/jbg.12443 31576623

[B53] Mao H. Xia Y. Tu Y. Wang C. Diao Q. (2017). Effects of various weaning times on growth performance, rumen fermentation and microbial population of yellow cattle calves. *Asian Austral. J. Anim. Sci.* 30 1557–1562. 10.5713/ajas.16.0981 28423879 PMC5666190

[B54] Min B. R. Gurung N. Shange R. Solaiman S. (2019). Potential role of rumen microbiota in altering average daily gain and feed efficiency in meat goats fed simple and mixed pastures using bacterial tag-encoded FLX amplicon pyrosequencing1. *J. Anim. Sci.* 97 3523–3534. 10.1093/jas/skz193 31214714 PMC6667257

[B55] Ndou S. P. Tun H. M. Kiarie E. Walsh M. C. Khafipour E. Nyachoti C. M. (2018). Dietary supplementation with flaxseed meal and oat hulls modulates intestinal histomorphometric characteristics, digesta- and mucosa-associated microbiota in pigs. *Sci. Rep.* 8:5880. 10.1038/s41598-018-24043-5 29651010 PMC5897541

[B56] North M. K. Zotte A. D. Hoffman L. C. (2019). Composition of rabbit caecal microbiota and the effects of dietary ouercetin supplementation and sex thereupon. *World Rabbit Sci.* 27 185–198.

[B57] O’Callaghan T. F. Ross R. P. Stanton C. Clarke G. (2016). The gut microbiome as a virtual endocrine organ with implications for farm and domestic animal endocrinology. *Domest Anim. Endocrinol.* 56(Suppl.), S44–S55. 10.1016/j.domaniend.2016.05.003 27345323

[B58] Oikonomou G. Teixeira A. G. Foditsch C. Bicalho M. L. Machado V. S. Bicalho R. C. (2013). Fecal microbial diversity in pre-weaned dairy calves as described by pyrosequencing of metagenomic 16S rDNA. Associations of Faecalibacterium species with health and growth. *PLoS One* 8:e63157. 10.1371/journal.pone.0063157 23646192 PMC3639981

[B59] Patra A. K. Geiger S. Schrapers K. T. Braun H. S. Gehlen H. Starke A. (2019). Effects of dietary menthol-rich bioactive lipid compounds on zootechnical traits, blood variables and gastrointestinal function in growing sheep. *J. Anim. Sci. Biotechnol.* 10:86. 10.1186/s40104-019-0398-6 31827785 PMC6886202

[B60] Piles M. Garcia-Tomas M. Rafel O. Ramon J. Ibanez-Escriche N. Varona L. (2007). Individual efficiency for the use of feed resources in rabbits. *J. Anim. Sci.* 85 2846–2853. 10.2527/jas.2006-218 17686894

[B61] Priyadarshini M. Kotlo K. U. Dudeja P. K. Layden B. T. (2018). Role of short chain fatty acid receptors in intestinal physiology and pathophysiology. *Compr. Physiol.* 8 1091–1115. 10.1002/cphy.c170050 29978895 PMC6058973

[B62] Qin J. Li Y. Cai Z. Li S. Zhu J. Zhang F. (2012). A metagenome-wide association study of gut microbiota in type 2 diabetes. *Nature* 490 55–60. 10.1038/nature11450 23023125

[B63] Quast C. Pruesse E. Yilmaz P. Gerken J. Schweer T. Yarza P. (2013). The SILVA ribosomal RNA gene database project: improved data processing and web-based tools. *Nucleic Acids Res.* 41 D590–D596. 10.1093/nar/gks1219 23193283 PMC3531112

[B64] Ramayo-Caldas Y. Mach N. Lepage P. Levenez F. Denis C. Lemonnier G. (2016). Phylogenetic network analysis applied to pig gut microbiota identifies an ecosystem structure linked with growth traits. *ISME J.* 10 2973–2977. 10.1038/ismej.2016.77 27177190 PMC5148198

[B65] Reddivari L. Veeramachaneni D. N. R. Walters W. A. Lozupone C. Palmer J. Hewage M. K. K. (2017). Perinatal bisphenol a exposure induces chronic inflammation in rabbit offspring via modulation of gut bacteria and their metabolites. *mSystems* 2:e00093-17. 10.1128/mSystems.00093-17 29034330 PMC5634791

[B66] Shannon P. Markiel A. Ozier O. Baliga N. S. Wang J. T. Ramage D. (2003). Cytoscape: a software environment for integrated models of biomolecular interaction networks. *Genome Res.* 13 2498–2504. 10.1101/gr.1239303 14597658 PMC403769

[B67] Shokryazdan P. Faseleh Jahromi M. Liang J. B. Ramasamy K. Sieo C. C. Ho Y. W. (2017). Effects of a *Lactobacillus salivarius* mixture on performance, intestinal health and serum lipids of broiler chickens. *PLoS One* 12:e0175959. 10.1371/journal.pone.0175959 28459856 PMC5411046

[B68] Sidhu M. van der Poorten D. (2017). The gut microbiome. *Aust. Fam. Phys.* 46 206–211. 28376573

[B69] Tran H. Anderson C. L. Bundy J. W. Fernando S. C. Miller P. S. Burkey T. E. (2018). Effects of spray-dried porcine plasma on fecal microbiota in nursery pigs. *J. Anim. Sci.* 96 1017–1031. 10.1093/jas/skx034 29385463 PMC6093563

[B70] Wang J. Liu Y. J. Yang Y. Z. Bao C. L. Cao Y. H. (2019). High level expression of an acidic thermostable xylanase in *Pichia pastoris* and its application in weaned piglets. *J. Anim. Sci.* 98:skz364. 10.1093/jas/skz364 31778535 PMC6986428

[B71] Wang Q. Fu W. Guo Y. Tang Y. Du H. Wang M. (2019). Drinking warm water improves growth performance and optimizes the gut microbiota in early postweaning rabbits during winter. *Animals* 9:346. 10.3390/ani9060346 31212853 PMC6616395

[B72] Wang T. J. Larson M. G. Vasan R. S. Cheng S. Rhee E. P. McCabe E. (2011). Metabolite profiles and the risk of developing diabetes. *Nat. Med.* 17 448–453. 10.1038/nm.2307 21423183 PMC3126616

[B73] Wang X. Liu Y. Li S. Pi D. Zhu H. Hou Y. (2015). Asparagine attenuates intestinal injury, improves energy status and inhibits AMP-activated protein kinase signalling pathways in weaned piglets challenged with *Escherichia coli* lipopolysaccharide. *Br. J. Nutr.* 114 553–565. 10.1017/S0007114515001877 26277838

[B74] Wang X. Tsai T. Deng F. Wei X. Chai J. Knapp J. (2019). Longitudinal investigation of the swine gut microbiome from birth to market reveals stage and growth performance associated bacteria. *Microbiome* 7:109. 10.1186/s40168-019-0721-7 31362781 PMC6664762

[B75] Wen C. Yan W. Sun C. Ji C. Zhou Q. Zhang D. (2019). The gut microbiota is largely independent of host genetics in regulating fat deposition in chickens. *ISME J.* 13 1422–1436. 10.1038/s41396-019-0367-2 30728470 PMC6775986

[B76] Xie J. Liu Y. Chen B. Zhang G. Ou S. Luo J. (2019). Ganoderma lucidum polysaccharide improves rat DSS-induced colitis by altering cecal microbiota and gene expression of colonic epithelial cells. *Food Nutr. Res.* 63:1559. 10.29219/fnr.v63.1559 30814921 PMC6387425

[B77] Xing S. C. Huang C. B. Mi J. D. Wu Y. B. Liao X. D. (2019). Bacillus coagulans R11 maintained intestinal villus health and decreased intestinal injury in lead-exposed mice by regulating the intestinal microbiota and influenced the function of faecal microRNAs. *Environ. Pollut.* 255(Pt 2), 113139. 10.1016/j.envpol.2019.113139 31563774

[B78] Yang H. Yang M. Fang S. Huang X. He M. Ke S. (2018). Evaluating the profound effect of gut microbiome on host appetite in pigs. *BMC Microbiol.* 18:215. 10.1186/s12866-018-1364-8 30547751 PMC6295093

[B79] Yang Z. Liao S. F. (2019). Physiological effects of dietary amino acids on gut health and functions of swine. *Front. Vet. Sci.* 6:169. 10.3389/fvets.2019.00169 31245390 PMC6579841

[B80] Yu M. Li Z. Chen W. Wang G. Cui Y. Ma X. (2019). Dietary supplementation with citrus extract altered the intestinal microbiota and microbial metabolite profiles and enhanced the mucosal immune homeostasis in yellow-feathered broilers. *Front. Microbiol.* 10:2662. 10.3389/fmicb.2019.02662 31849855 PMC6887900

[B81] Yu X. Avall-Jaaskelainen S. Koort J. Lindholm A. Rintahaka J. von Ossowski I. (2017). A comparative characterization of different host-sourced *Lactobacillus ruminis* strains and their adhesive, inhibitory, and immunomodulating functions. *Front. Microbiol.* 8:657. 10.3389/fmicb.2017.00657 28450859 PMC5390032

[B82] Zeng B. Han S. Wang P. Wen B. Jian W. Guo W. (2015). The bacterial communities associated with fecal types and body weight of rex rabbits. *Sci. Rep.* 5:9342. 10.1038/srep09342 25791609 PMC4366860

[B83] Zhang K. Li B. Guo M. Liu G. Yang Y. Wang X. (2019). Maturation of the goat rumen microbiota involves three stages of microbial colonization. *Animals* 9:1028. 10.3390/ani9121028 31775375 PMC6941170

[B84] Zhang L. Wu W. Lee Y. K. Xie J. Zhang H. (2018). Spatial heterogeneity and co-occurrence of mucosal and luminal microbiome across swine intestinal tract. *Front. Microbiol.* 9:48. 10.3389/fmicb.2018.00048 29472900 PMC5810300

[B85] Zhang Q. Wu Y. Wang J. Wu G. Long W. Xue Z. (2016). Accelerated dysbiosis of gut microbiota during aggravation of DSS-induced colitis by a butyrate-producing bacterium. *Sci. Rep.* 6:27572. 10.1038/srep27572 27264309 PMC4893749

[B86] Zhang S. Song J. Deng Z. Cheng L. Tian M. Guan W. (2017). Effects of combined alpha-galactosidase and xylanase supplementation on nutrient digestibility and growth performance in growing pigs. *Arch. Anim. Nutr.* 71 441–454. 10.1080/1745039X.2017.1389217 29110578

[B87] Zhang T. Li Q. Cheng L. Buch H. Zhang F. (2019). Akkermansia muciniphila is a promising probiotic. *Microb. Biotechnol.* 12 1109–1125. 10.1111/1751-7915.13410 31006995 PMC6801136

[B88] Zhu W. Lomsadze A. Borodovsky M. (2010). Ab initio gene identification in metagenomic sequences. *Nucleic Acids Res.* 38:e132. 10.1093/nar/gkq275 20403810 PMC2896542

