# Supplementary figures and images for: Effects of Gut Microbiome and Short-Chain Fatty Acids (SCFAs) on Finishing Weight of Meat Rabbits
Source: Front Microbiol. 2020 Aug 11;11:1835. doi: 10.3389/fmicb.2020.01835 (PMC7431612; doi:10.3389/fmicb.2020.01835)

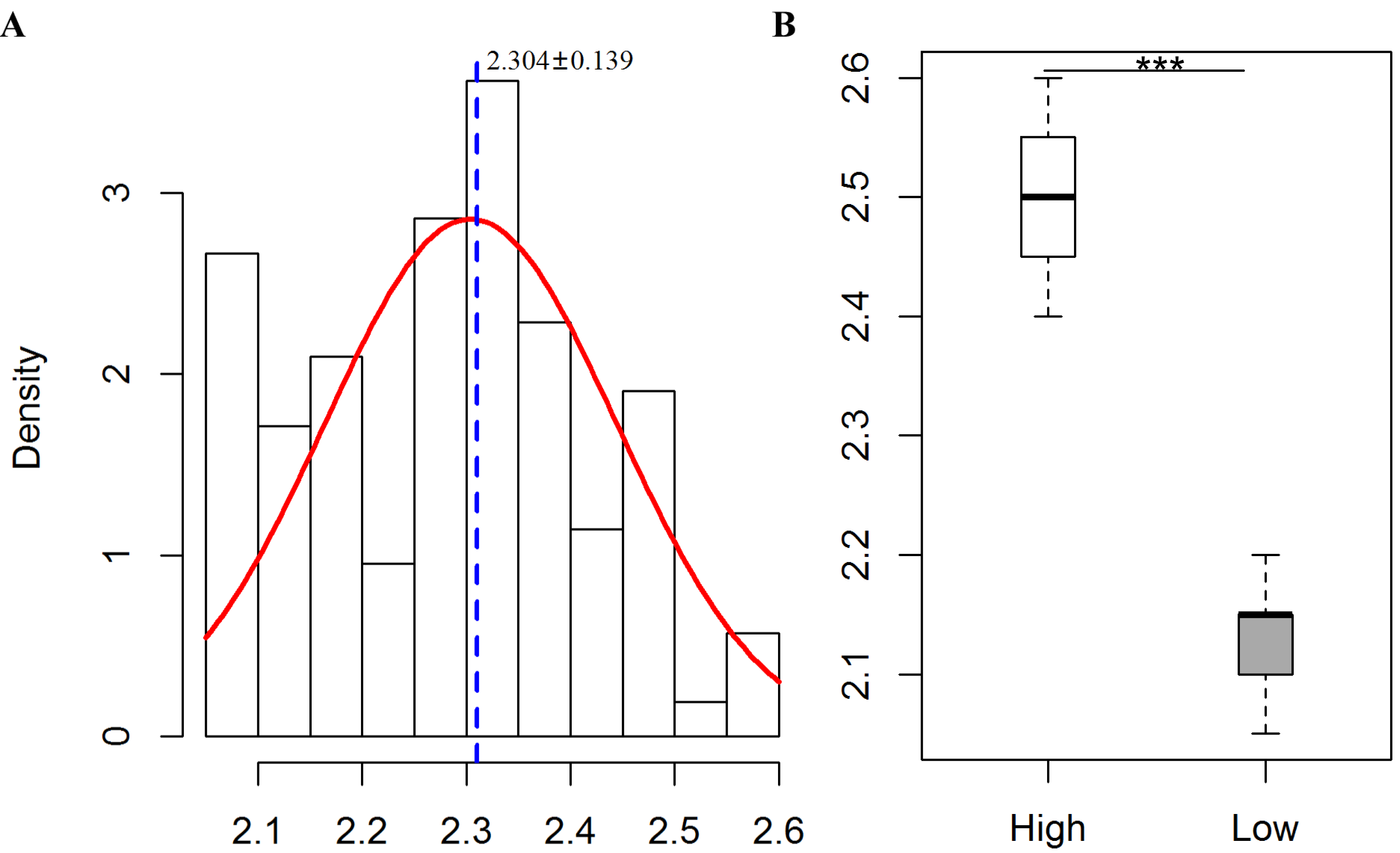

Supplement: FIGURE S1 — Finishing weight phenotypic values of all rabbits (A) and high and low individuals (B). [file Image_1.TIFF]

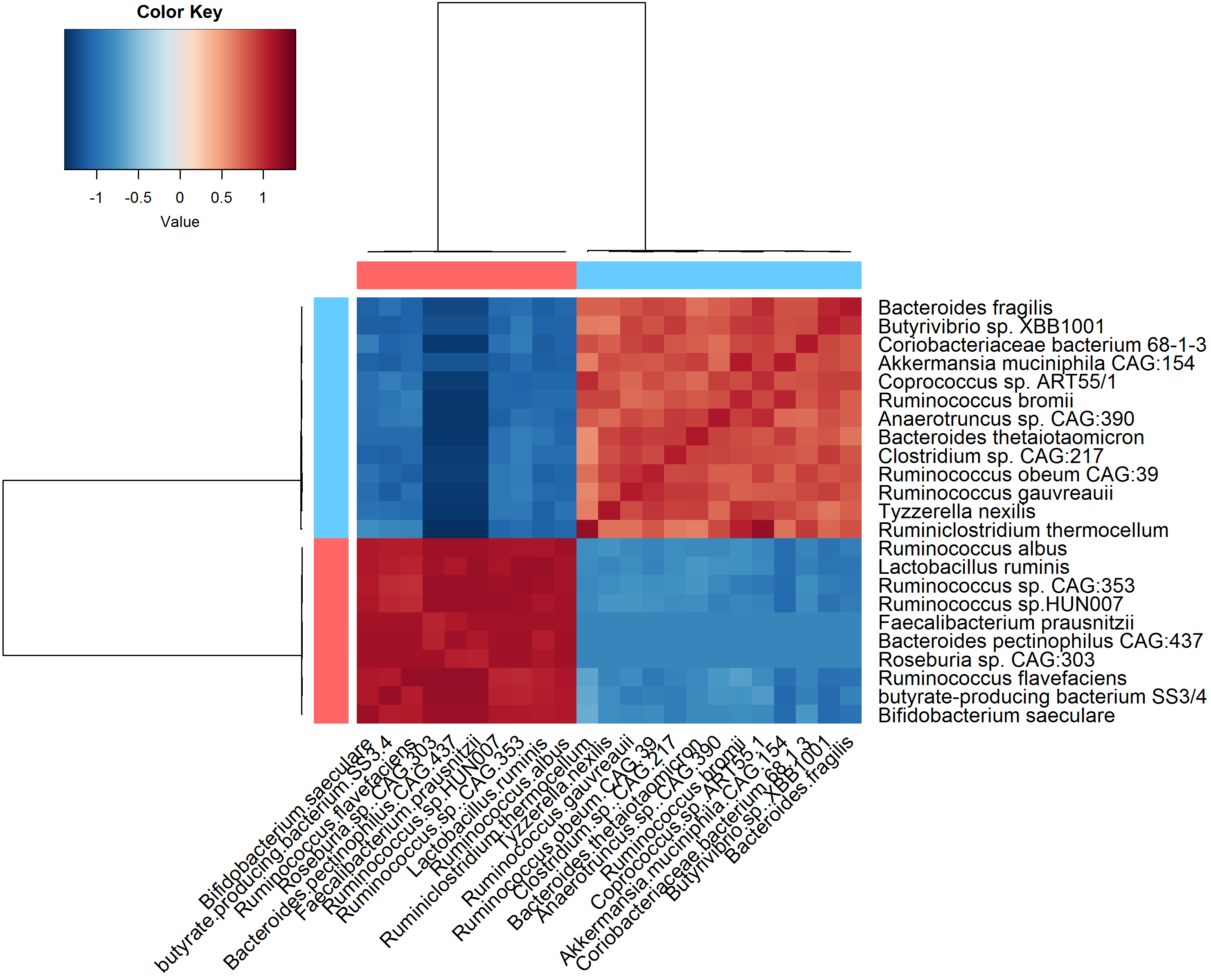

Supplement: FIGURE S2 — The finishing weight associated species formed two clusters using Ward clustering algorithm. [file Image_2.TIFF]

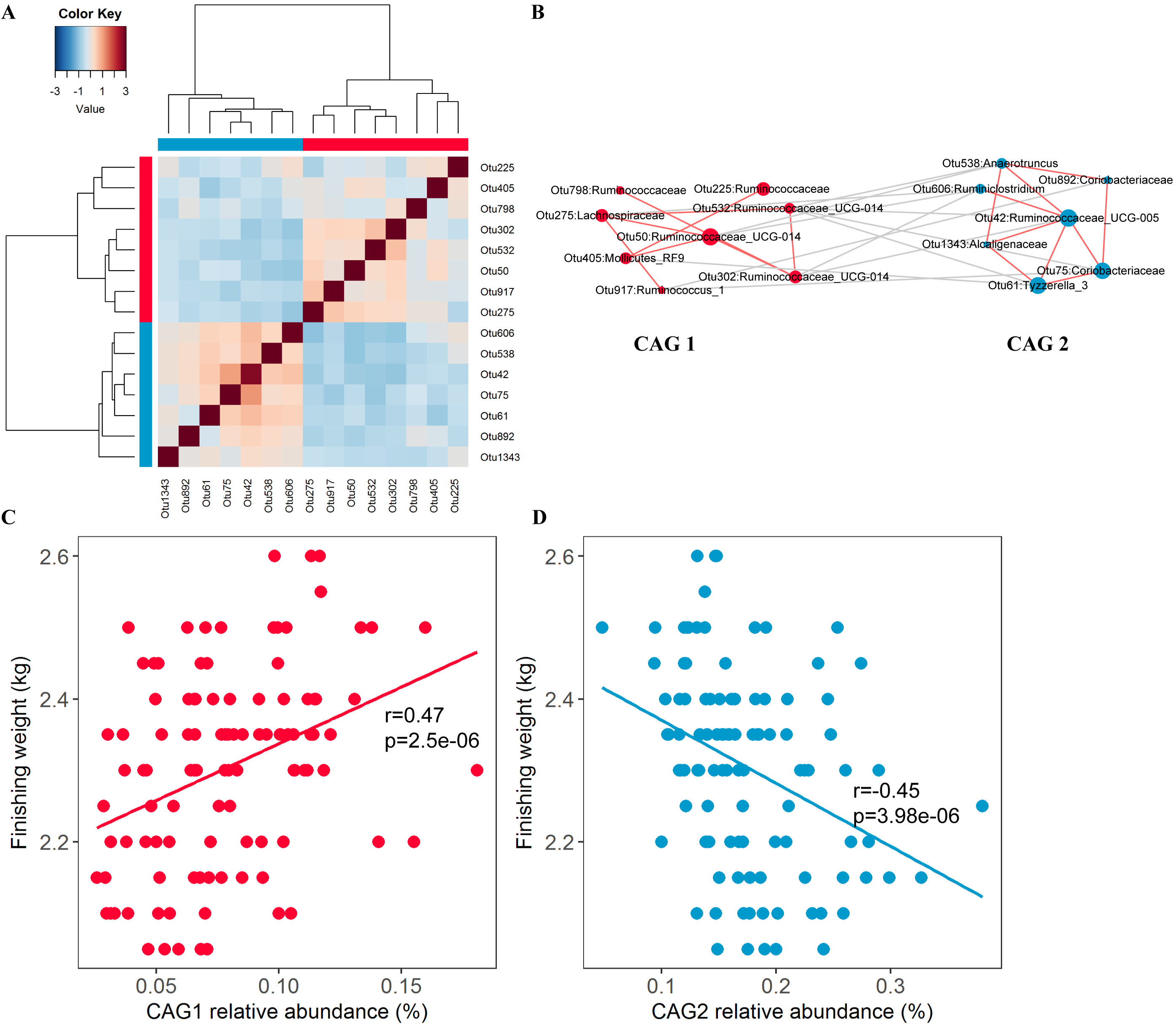

Supplement: FIGURE S3 — (A,B) Constructions of CAGs and interactions network by using the finishing weight associated OTUs. (C,D) Associations between CAGs and finishing weight. [file Image_3.TIFF]
